# MUC1: The First Respiratory Mucin with an Anti-Inflammatory Function

**DOI:** 10.3390/jcm6120110

**Published:** 2017-11-29

**Authors:** Kosuke Kato, Erik P. Lillehoj, Wenju Lu, Kwang Chul Kim

**Affiliations:** 1 Department of Otolaryngology, University of Arizona College of Medicine, Tucson, AZ 85724, USA; kosukekato@oto.arizona.edu; 2 Department of Pediatrics, University of Maryland School of Medicine, Baltimore, MD 21201, USA; elillehoj@peds.umaryland.edu; 3 Department of Medicine, The First Hospital of Guangzhou Medical University, Guangzhou 510120, China; wlu92@yahoo.com; 4 Departments of Physiology and Medicine, University of Arizona College of Medicine, Tucson, AZ 85724, USA

**Keywords:** MUC1, membrane-tethered mucin, lung, inflammation, *Pseudomonas aeruginosa*, Toll-like receptor

## Abstract

MUC1 is a membrane-bound mucin expressed on the apical surfaces of most mucosal epithelial cells. In normal lung epithelia, MUC1 is a binding site for *Pseudomonas aeruginosa*, an opportunistic human pathogen of great clinical importance. It has now been established that MUC1 also serves an anti-inflammatory role in the airways that is initiated late in the course of a bacterial infection and is mediated through inhibition of Toll-like receptor (TLR) signaling. MUC1 expression was initially shown to interfere with TLR5 signaling in response to *P. aeruginosa* flagellin, but has since been extended to other TLRs. These new findings point to an immunomodulatory role for MUC1 during *P. aeruginosa* lung infection, particularly during the resolution phase of inflammation. This review briefly summarizes the recent characterization of MUC1’s anti-inflammatory properties in both the respiratory tract and extrapulmonary tissues.

## 1. Introduction

Mucus lining the airway lumen serves as a major protective barrier for the lung. Although many airborne particles and toxic chemicals are trapped in the mucus layer and continuously cleared by the mucociliary escalator, some have evolved mechanisms to circumvent this barrier. Any abnormalities in the quantity and/or quality of mucus can cause serious pulmonary complications, often leading to the death of patients with diseases such as cystic fibrosis, bronchiectasis, chronic obstructive pulmonary disease, and asthma. Mucins are the major glycoproteins present in mucus, and their presence contributes to the viscoelastic properties of mucus. Twenty-one mucin genes have been identified in humans, 14 of which are expressed in the respiratory tract [1]. The translated products of these mucin genes are broadly classified as either gel-forming, secreted, or membrane-tethered mucins. All mucin proteins are heavily glycosylated, primarily through the post-translational attachment of O-linked glycans, and all mucins contain variable numbers of tandemly repeated amino acid sequences with a high content of serine, threonine, and proline residues. Among the membrane-tethered mucins in the respiratory tract—primarily MUC1, MUC4, and MUC16—MUC1 has been the best characterized. MUC1 was originally described as an adhesion site for *Pseudomonas aeruginosa* on the surface of airway epithelial cells [2,3,4], but more recent evidence indicates that it also plays a central, anti-inflammatory role following activation of host inflammation in response to a variety of infectious insults [5,6,7,8,9,10]. In this review, we briefly highlight the characteristics of MUC1 as an anti-inflammatory respiratory mucin.

## 2. Structure of the MUC1 Glycoprotein

**Figure 1:**
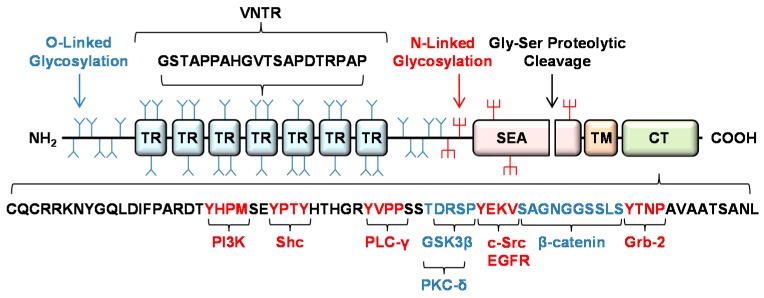
Schematic diagram of the MUC1 glycoprotein. Proceeding from the NH_2_-terminus to the COOH-terminus, MUC1 consists of heavily O-glycosylated ectodomain containing a variable number of tandem repeat (VNTR) region, a SEA domain with a glycine-serine (Gly-Ser) proteolytic cleavage site, a single pass transmembrane (TM) domain, and a cytoplasmic tail (CT). The amino acid sequence of a single TR is indicated. Five potential sites for N-linked glycosylation are present in the MUC1 ectodomain and SEA domain. The sequence of the 72-amino acid MUC1-CT is illustrated with potential binding sites for various kinases and adapter proteins at tyrosine-containing (red) and serine/threonine-containing (blue) sequence motifs.

The MUC1 protein consists of a large extracellular domain, a single-pass transmembrane region, and an intracellular cytoplasmic tail (CT) (Figure 1). The extracellular region of MUC1 is heavily glycosylated, primarily through O-linked glycans within its 20-amino acid variable number of tandem repeat (VNTR) region, and serves as a binding site for *Pseudomonas aeruginosa* [2,3,4,5]. *P. aeruginosa* is the most frequently isolated bacterial pathogen from the respiratory tract of intensive care unit patients and contributes to the decline in lung function in patients with cystic fibrosis, bronchiectasis, and chronic obstructive pulmonary disease [11]. The MUC1 protein is autoproteolytically cleaved at a glycine–serine peptide bond located within the SEA (sea urchin sperm protein, enterokinase, agrin) region of its ectodomain, an ~120-amino acid region that is highly conserved in several mucin-like glycoproteins [12,13,14,15]. This autoproteolytic cleavage generates a protein heterodimer with the two subunits remaining tightly associated on the cell surface [16,17]. Independently, the MUC1 ectodomain is cleaved by three proteases, tumor necrosis factor (TNF) converting enzyme (TACE), matrix metalloproteinase-14, and γ-secretase [18,19,20]. The action of these MUC1 sheddases allows the majority of its NH_2_-terminal ectodomain to be released from the cell surface and act as a soluble decoy to block *P. aeruginosa* adhesion to cell-associated MUC1 [21]. At the COOH-terminal portion of the protein, the 72-amino acid MUC1-CT contains 7 evolutionally conserved tyrosine residues, the majority of which are phosphorylated and serve as potential binding sites for kinases and adapter proteins involved in signal transduction [22,23,24,25,26]. These include phosphoinositide 3-kinase (PI3K) (Y^20^HPM), Src homology 2 domain containing protein (Shc) (YPTY^29^), phospholipase C-γ (PLC-γ) (Y^35^VPP), epidermal growth factor receptor (EGFR), c-Src (Y^46^EKV), and growth factor receptor-bound protein 2 (Grb2) (Y^60^TNP). Amino acids are indicated by the single letter code. Tyrosine residues are numbered beginning with the first amino acid of the MUC1-CT. Additional sites within the MUC1-CT serve as binding sites for other cytosolic signaling molecules, including protein kinase C-δ (PKC-δ) (T^41^DRS), glycogen synthase kinase 3β (GSK3β) (D^42^RSP), and β-catenin (S^50^AGNGGSSL) [23,27,28]. While these features of MUC1 have suggested a cytokine/growth factor receptor-like structure, the MUC1-CT is not capable of autophosphorylation [29]. For more details about MUC1 structure and signaling, see the reviews by Albrecht and Carraway [30], Kufe [31], Mehla and Singh [32], Haddon and Hugh [33], and Hanson and Hollingsworth [34].

## 3. Expression of MUC1 in the Respiratory Tract

*MUC1* (MUC1 in humans, *Muc1* in animals) was the first mucin gene to be cloned [35,36]. Prior to *MUC1* gene cloning, we reported the presence of mucin-like glycoproteins on the surface of hamster airway epithelial cells that were released from the cell surface by neutrophil elastase, a potent mucin secretagogue [37]. This elastase-releasable airway mucin was subsequently identified as hamster Muc1 based on its homology to human MUC1 [38,39]. In human respiratory epithelia, *MUC1* mRNA was first detected using in vitro cultures of nasal and bronchial epithelial cells [40] and MUC1 protein was identified in ex vivo human lung explants [41]. It is now widely appreciated that MUC1/Muc1 is expressed on the surface of most mucosal epithelia—including the respiratory, gastrointestinal, and genitourinary tracts—as well as the mammary and salivary glands [5,42,43], in addition to cells of the hematopoietic system, including CD4^+^, CD8^+^, and Th17 lymphocytes, dendritic cells, monocytes, and macrophages [44,45,46,47,48,49,50,51]. In the upper airways, MUC1 protein has been localized to nasal, pharyngeal, and laryngeal epithelial cells [40,52,53], while in the lower airways, MUC1 is expressed by bronchial, tracheal, and alveolar epithelial cells [40,54,55]. In addition to mucosal epithelial cells and hematopoietic cells, the expression of MUC1 has also been reported in human corneal [56] and human umbilical vein endothelial cells [57], and human skin fibroblasts [58]. Whether or not MUC1 is also expressed in these cell types in the lung and contributes to airway inflammation remains to be investigated.

## 4. MUC1 Is an Airway Epithelial Cell Binding Site for *Pseudomonas aeruginosa*

We initially proposed that MUC1 on the cell surface was an adhesion site for *P. aeruginosa* based on its receptor-like structure [29] and the ability of *P. aeruginosa* to bind to secreted mucins [59]. To test this hypothesis, Chinese hamster ovary (CHO) cells, which do not express endogenous *Muc1*, were stably transfected with a hamster *Muc1* cDNA, and binding of *P. aeruginosa* to the CHO-Muc1 cells examined. CHO-Muc1 cells exhibited approximately two-fold increased bacterial adhesion compared with cells transfected with the empty vector [2,3]. Further, bacterial adhesion to CHO-Muc1 cells was completely abolished following proteolytic release of Muc1 from the cell surface with neutrophil elastase or by deletion of the Muc1 ectodomain. More recently, *P. aeruginosa* adhesion to human airway epithelial cells endogenously expressing MUC1 has been demonstrated [4]. Knock down of *MUC1* gene expression in human bronchial or alveolar epithelial cells by RNA interference significantly reduced bacterial adhesion to the cells compared with a nontargeting control small interfering RNA (siRNA). Further, desialylation of the MUC1 ectodomain by ectopic overexpression of the NEU1 sialidase increased *P. aeruginosa* adhesion to airway epithelial cells compared with the vector control, suggesting that sialic acid residues of MUC1 glycans mask bacterial binding sites [19,60]. The molecular adhesin of *P. aeruginosa* utilized to bind to MUC1/Muc1 was identified as flagellin, the major structural protein of the bacterial flagellar filament [61]. Since bacterial flagellin also engages TLR5 on airway epithelial cells [62], it was of interest to assess the contribution of TLR5 to *P. aeruginosa* adhesion. However, when human embryonic kidney 293 (HEK293) cells, which normally do not express either MUC1 or TLR5, were separately transfected for MUC1 or TLR5 expression and assayed for *P. aeruginosa* adhesion, only MUC1-expressing HEK293 cells—but not TLR5-expressiong cells—exhibited greater bacterial adhesion compared with cells transfected with the empty vector control [21].

## 5. Inhibition of Airway Inflammation by MUC1

Bacterial adhesion to host cell surface receptors often is the initial step in colonization, subsequently leading to activation of intracellular signaling pathways that culminate in the expression of proinflammatory mediators in response to the invading pathogen [63,64]. Because *P. aeruginosa* stimulation of airway epithelial cells in vitro activates a proinflammatory extracellular signal-regulated kinase 1/2 (ERK1/2) signaling cascade [60,65], we expected that *Muc1* knockout mice, generated in 1995 by Dr. Sandra J. Gendler [66], would exhibit diminished airway inflammation and increased bacterial colonization following *P. aeruginosa* lung infection. Surprisingly, approximately 10-fold fewer numbers of bacteria were recovered from the lungs of *Muc1^−/−^* mice at 16 h following acute lung infection with a single inoculum of *P. aeruginosa* compared with *Muc1^+/+^* mice [67]. In addition, *Muc1^−/−^* mice also had increased post-infection levels of proinflammatory keratinocyte chemoattractant (KC) and TNF in their bronchoalveolar fluid (BALF), and greater levels of BALF leukocytes compared with *Muc1^+/+^* mice. BALF KC and TNF levels also were increased in *Muc1^−/−^* mice following airway instillation of flagellin purified from *P. aeruginosa*, indicating that Muc1 expression counter-regulated TLR5-driven inflammation [67]. Further, primary tracheal epithelial cells and alveolar macrophages, both isolated from *Muc1^−/−^* mice and stimulated in vitro with *P. aeruginosa* flagellin, produced more KC and TNF, respectively, compared with the corresponding *Muc1^+/+^* cells. In a second mouse model of chronic *P. aeruginosa* lung infection where animals were challenged 4 times with bacteria over 10 days, *Muc1^−/−^* mice exhibited greater post-infection body weight loss, greater numbers of alveolar macrophages, and increased alveolar airspace enlargement compared with *Muc1^+/+^* in mice [68]. To extend these results to a human system, we showed that knockdown of MUC1 expression by RNA interference in primary normal human bronchial epithelial (NHBE) cells enhanced flagellin-induced interleukin-8 (IL-8) production [67].

The anti-inflammatory effects of MUC1 have also been extended to TLRs other than TLR5. Silencing *MUC1* gene expression in BEAS-2B human airway epithelial cells by siRNA transfection enhanced IL-8 production following stimulation with the TLR4 agonist, lipopolysaccharide (LPS), compared with MUC1-expressing cells [69]. In vitro cultures of peritoneal and alveolar macrophages from *Muc1^−/−^* mice produced greater levels of TNF following stimulation with the TLR agonists Pam3Cys (TLR2), poly(I:C) (TLR3), LPS (TLR4), loxoribine (TLR7), and CpG DNA (TLR9) compared with cells from *Muc1^+/+^* mice [70]. HEK293 cells transfected with plasmids encoding for TLR2, TLR3, TLR4, TLR5, TLR7, or TLR9 in the absence of MUC1 expression exhibited greater nuclear factor-κB (NF-κB) activation in response to stimulation with the respective TLR agonists compared with cells co-transfected with the individual TLRs plus MUC1. These same studies mapped the inhibitory effect of MUC1 on TLR-driven macrophage activation to the MUC1-CT, and not the MUC1 ectodomain [70].

Other studies have evaluated the anti-inflammatory effects of MUC1/Muc1 in the host response to viral and bacterial airway pathogens other than *P. aeruginosa*. MUC1 expression limited the severity of influenza A virus infection in both human and mouse airway epithelial cells, although the mechanism was hypothesized not to be mediated through the MUC1-CT, but rather to involve virus binding to the MUC1 ectodomain, blocking subsequent host cell infection [6]. MUC1 not only binds to influenza virus and inhibits infection, but also in the absence of Muc1 expression, an enhanced inflammatory disease was experienced by the virus-infected mice. *Muc1^−/−^* mice intranasally infected with *Streptococcus pneumoniae* had greater lung inflammation with increased recruitment of monocytes and macrophages compared with *Muc1^+/+^* mice [7]. In our own studies, in vitro cultures of human A549 airway epithelial cells transfected with *MUC1*-targeting siRNAs and stimulated with either respiratory syncytial virus (RSV) or nontypeable *Haemophilus influenzae* (NTHi) produced greater levels of TNF and IL-8 compared with cells transfected with nontargeting control siRNAs [8,9]. Conversely, overexpression of MUC1 in these cells reduced cytokine/chemokine levels compared with empty vector controls. Interestingly, TNF release by MUC1-expressing cells following stimulation by either RSV or NTHi preceded upregulation of MUC1 protein levels, which was completely inhibited by pretreatment with a soluble TNF receptor (TNFR). Taken together with prior studies, these results suggested a theoretical feedback loop model whereby airway pathogens activate TLRs on airway epithelial cells, leading to an early increase in NF-κB activation, and TNF and IL-8 production, which subsequently upregulates MUC1 expression, leading to later suppression of TLR activation and decreased cytokine and chemokine production. To test this hypothetical model, *Muc1^−/−^* and *TNFR^−/−^* mice, and their wild type littermates, were infected with *P. aeruginosa* and TNF and MUC1 levels in the lungs were monitored over time and compared with uninfected mice [71]. In *Muc1^+/+^* mouse lungs, Muc1 levels were low both in uninfected and infected lungs at early time points following infection. Muc1 levels increased post-infection, reaching maximum levels after two days which were maintained even seven days post-infection. However, *TNFR^−/−^* mice failed to upregulate Muc1 expression following *P. aeruginosa* infection, and both *Muc1^−/−^* and *TNFR^−/−^* mice were unable to spontaneously resolve *P. aeruginosa*-induced lung inflammation.

## 6. TNF Increases MUC1 Expression through a Mitogen-Activated Protein Kinase (MAPK) → Sp1 Signaling Pathway

TNF is a proinflammatory cytokine produced by multiple cell types in response to a wide variety of infectious and noninfectious stimuli [72]. TNF binds to its cognate receptor, TNFR, leading to the activation of intracellular signaling cascades and culminating in a diverse array of biological responses. TNF upregulates MUC1 expression in airway, breast, prostate, and uterine epithelial cells [73,74,75,76]. In airway epithelia, TNF increased *MUC1* mRNA and protein levels, but did not alter *MUC1* transcript stability, implying increased *MUC1* gene transcription [77]. TNF stimulation of these same cells increased *MUC1* gene promoter activity in an in vitro luciferase assay, and pharmacological blockade of the MAPK, MEK1/2, reduced TNF-induced *MUC1* promoter activation. Further, while TNF stimulation increased ERK1/2 phosphorylation, only ectopic overexpression of a dominant negative (DN) ERK1, but not DN ERK2, diminished TNF-induced *MUC1* promoter activation. An anti-TNFR blocking antibody inhibited TNF-stimulated ERK1/2 phosphorylation. Finally, *MUC1* promoter activation by TNF was blocked by mithramycin A, an inhibitor of the Sp1 transcription factor, and by deletion of a putative Sp1 binding site in the *MUC1* promoter, and in chromatin immunoprecipitation assays, TNF stimulation of airway epithelial cells increased binding of Sp1 to the *MUC1* promoter. These results indicated that TNF increased MUC1 expression in airway epithelial cells through a TNFR → MEK1/2 → ERK1 → Sp1 signaling pathway [77].

## 7. Role of Peroxisome Proliferator-Activated Receptor-γ (PPAR-γ) in the Anti-Inflammatory Effects of MUC1

PPAR-γ is a nuclear receptor with anti-inflammatory properties [78]. Because the human and mouse *MUC1/Muc1* gene promoters both contain a putative PPAR-γ-binding site and because PPAR-γ stimulation of mouse trophoblast cells increased Muc1 protein expression [79], we hypothesized that PPAR-γ might mediate its anti-inflammatory effects, in part, through increased MUC1/Muc1 expression. In human airway epithelial cells, the PPAR-γ agonist, troglitazone (TGN), diminished *P. aeruginosa*-stimulated IL-8 production through a MUC1-dependent mechanisms compared with vehicle controls [80]. In addition, TGN stimulation of these cells increased PPAR-γ-binding to the *MUC1* gene promoter and increased *MUC1* mRNA and protein levels compared with the vehicle control. In a subsequent study, PPAR-γ was identified upstream of MUC1 in an anti-inflammatory pathway suppressing *Helicobacter pylori*-stimulated IL-8 production by gastric epithelial cells [81]. These reports [80,81] were the first to demonstrate a functional relationship between MUC1 and PPAR-γ, two anti-inflammatory molecules expressed in mucosal epithelia.

## 8. The MUC1-CT Associates with TLR5 to Inhibit Recruitment of Adaptor Proteins

To determine the molecular mechanism through which MUC1 might counter-regulate TLR-dependent airway inflammation, experiments were performed examining protein–protein interactions between MUC1-CT, TLR5, and the myeloid differentiation primary response gene 88 (MyD88) protein, an adaptor molecule that binds to the cytosolic TIR domain of the TLR5 to initiate intracellular signaling [82]. Overexpression of MUC1 in HEK293 cells reduced flagellin-stimulated, TLR5-driven activation of ERK1/2, p38, and NF-κB compared with the empty vector controls [83]. However, MAPK and NF-κB activation were not affected in MUC1-overexpressing cells in response to TNF stimulation. Because MAPK and NF-κB activation downstream of both TLR5 and TNFR require prior activation of TGF-β-activated kinase 1 (TAK1), and because the signaling pathways leading to activation of TAK1 by these two receptors are distinct, these results suggested that cross-talk between MUC1 and TLR5 signaling occurs upstream of TAK1, i.e., either at the level of either MyD88, interleukin-1 receptor-associated kinase 1 (IRAK1), or TNFR-associated factor 6 (TRAF6). Overexpression of MyD88, but not IRAK1 nor TRAF6, restored flagellin-stimulated NF-κB activation in the presence of MUC1 expression compared with the empty vector control [83]. Importantly, *P. aeruginosa* stimulated the association of MUC1-CT with TLR5 in co-immunoprecipitation assays, and MUC1 overexpression blocked flagellin-induced TLR5/MyD88 protein–protein interaction, both compared with the respective controls. Together, these results indicated that *P. aeruginosa* stimulation of human airway epithelial cells promoted MUC1-CT/TLR5 protein interaction to block recruitment of MyD88 to TLR5. Because MUC1 expression counter-regulates TLR3 activation in response to poly(I:C) [70], we asked whether the MUC1-CT might also interfere with recruitment of the TLR3 adaptor, TIR-domain-containing adapter-inducing interferon-β (TRIF), to TLR3. MUC1 overexpressing HEK293 cells exhibited reduced poly(I:C)-stimulated TLR3/TRIF co-immunoprecipitation, suggesting that like the TLR5/MyD88 interaction, the MUC1-CT blocked ligand-induced TLR3/TRIF protein association [84].

## 9. EGFR Activation Increases MUC1-CT Association with TLR5

**Figure 2:**
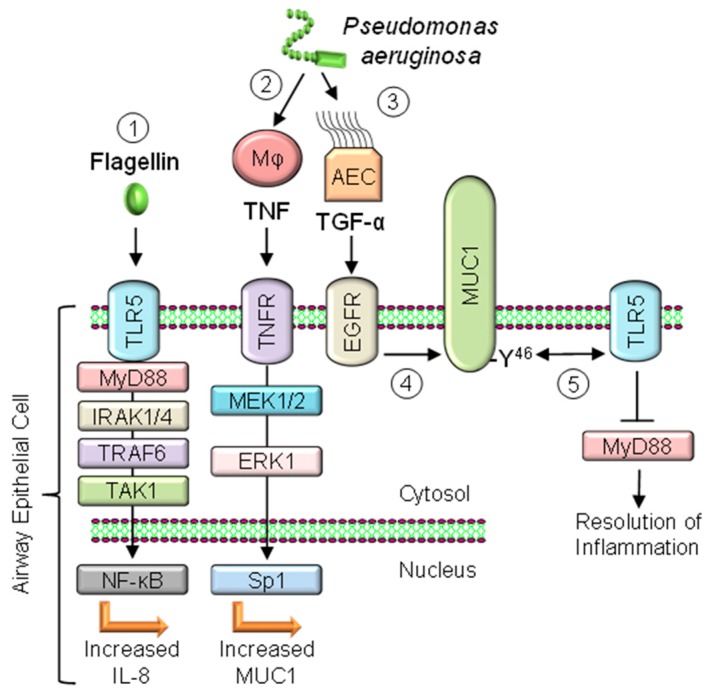
Hypothetical model for the proposed anti-inflammatory role of MUC1 in response to *P. aeruginosa* lung infection. (1) Flagellin engagement of TLR5 triggers a canonical MyD88 → IRAK1/4 → TRAF6 → TAK1 → NF-κB-signaling pathway to generate an early proinflammatory response with increased IL-8 production; (2) Inhaled *P. aeruginosa* activates alveolar macrophages (Mφ) to release TNF, which increases MUC1 expression by airway epithelial cells (AEC) through a TNFR → MEK1/2 → ERK1 → Sp1 pathway; (3) *P. aeruginosa* stimulation of AEC drives TGF-α release; (4) TGF-α binding to EGFR stimulates phosphorylation of the MUC1-CT at its tyrosine^46^ residue; (5) Phosphorylated MUC1-CT binds to the intracellular domain of TLR5, thereby blocking recruitment of MyD88 to TLR5, inhibiting inflammatory signaling, and contributing to the later resolution of inflammation.

The MUC1-CT contains multiple tyrosine residues as potential binding sites for protein kinases and signaling proteins, including EGFR [24]. In A549 lung epithelial cells, activation of EGFR by its ligand, transforming growth factor-α (TGF-α), stimulated phosphorylation of the MUC1-CT tyrosine^46^ residue and increased MUC1-CT/TLR5 co-immunoprecipitation [83]. In a subsequent study, stimulation of primary NHBE cells with *P. aeruginosa* enhanced MUC1-CT tyrosine phosphorylation and increased MUC1-CT/TLR5 and MUC1-CT/EGFR co-immunoprecipitation [85]. *P. aeruginosa* and flagellin stimulated release of TGF-α from NHBE cells, and both agonists elicited TNF release from primary macrophages, but not from the NHBE cells. Further, exogenous TNF, but not *P. aeruginosa*, upregulated MUC1 expression by NHBE cells. Based on these collective results, and combined with the established TLR5 signaling pathway [82], we proposed a model for the roles of TGF-α, TNF, EGFR, and MUC1 in the response of alveolar macrophages and airway epithelial cells to *P. aeruginosa* (Figure 2).

In this model, flagellin from inhaled bacteria activates a canonical TLR5 → MyD88 → IRAK1/4 → TRAF6 → TAK1 → NF-κB pathway leading to increased IL-8 production and recruitment of neutrophils to the airways and driving a proinflammatory response. *P. aeruginosa* also stimulates alveolar macrophages to release TNF, which increases MUC1 expression by airway epithelial cells in a paracrine manner. *P. aeruginosa* stimulation of macrophages also drives TGF-α release, leading to sequential EGFR activation, MUC1-CT tyrosine^46^ phosphorylation, and MUC1-CT/TLR5 protein association, thereby blocking MyD88 recruitment to TLR5 and inhibiting airway inflammation [85].

## 10. Additional Role of Airway Macrophages in the Anti-Inflammatory Effects of MUC1

Alveolar macrophages play a critical role in the host response against inhaled bacterial pathogens [86]. Classically-activated (M1) macrophages express high levels of proinflammatory cytokines and mediate antimicrobial host defense, while alternatively-activated (M2) macrophages are associated with the resolution of inflammation and tissue repair. We asked whether M1 and/or M2 macrophages express MUC1. *MUC1* mRNA and protein were expressed at greater levels in M1 human and mouse alveolar macrophages compared with M2 macrophages [51]. Further, *P. aeruginosa* stimulation increases MUC1-EC shedding from human M1 macrophages in a TACE-dependent manner, and MUC1/Muc1 expression by human or mouse macrophages decreased *P. aeruginosa* phagocytosis and reduced bacteria-stimulated reactive oxygen species (ROS) production and TNF release compared with MUC1/Muc1-expressing cells. While Muc1 deficiency was associated with increased phagocytosis of *P. aeruginosa*, macrophages from *Muc1^−/−^* mice exhibited a reduced capacity to phagocytose *S. pneumoniae* indicating diverse and bacterial-specific effects [7]. As such, MUC1 protection against severe pneumococcal disease may potentially be mediated by facilitating macrophage phagocytosis. Given that *P. aeruginosa* binds to MUC1/Muc1 through its flagella [61], it is possible that this difference in phagocytosis may be related, in part, to the fact that *S. pneumoniae* is non-flagellated. We next investigated the mechanism through which *P. aeruginosa* regulates MUC1 expression by macrophages [87]. *P. aeruginosa* stimulation of human macrophages increased MUC1 expression both at the transcriptional and protein levels in a dose-dependent manner. Both *P. aeruginosa*- and LPS-induced MUC1 expression in these cells were significantly diminished by a TLR4 antagonist. Finally, LPS-stimulated MUC1 expression by macrophages was diminished by an inhibitor of the p38 MAPK, but not by inhibitors of ERK1/2, c-Jun N-terminal kinases 1/2 (JNK1/2), or NF-κB. We concluded that *P. aeruginosa*-stimulated MUC1 expression in human macrophages is regulated through a TLR4 → p38 MAPK pathway.

## 11. Anti-Inflammatory Effects of MUC1 Outside of the Airways

Because MUC1 is expressed by most mucosal epithelia as well as hematopoietic cells [5,42,43], it is not surprising that its anti-inflammatory properties that have been described in the airways also have been reported in extrapulmonary tissues. In the hematopoietic system, antibody-based cross-linking of MUC1 on the T cell surface was associated with reduced cell proliferation, and diminished IL-2 and granulocyte-macrophage colony-stimulating factor (GM-CSF) production [45]. Muc1 was shown to function in a negative feedback pathway that prevented an excessive Th17 cell response in a mouse model of colitis [47]. *Muc1^−/−^* mice exhibited increased Th1/Th17 responses and greater pathology in a mouse model of experimental autoimmune encephalomyelitis [88] and exacerbated pulmonary fibrosis in a mouse model of silicosis [89]. *Muc1^−/−^* mice were predisposed to developing bacterial conjunctivitis due to infections by *Staphylococcus*, *Streptococcus*, or *Corynebacterium* compared with *Muc1^+/+^* mice [10]. Two *MUC1* mRNA isoforms, *MUC1/A* and *MUC1/B*, that are differentially expressed in dry eye disease [90], were studied in transfected COS-7 cells for their counter-regulatory effects on TNF-induced cytokine responses [91]. MUC1/A, which contains an additional nine amino acid segments within the MUC1 ectodomain and is expressed at low levels in dry eye disease patients [90], had less anti-inflammatory activity compared with MUC1/B [91]. Finally, compared with *Muc1^+/+^* mice, *Muc1^−/−^* mice expressed reduced levels of transcripts for IL-6 and TNF in the corneal epithelium following exposure of eyes to the TLR2 and TLR5 agonists, heat-killed *Listeria monocytogenes* and flagellin, respectively [92].

In the gastric mucosa, *Muc1^−/−^* mice had increased macrophage inflammatory protein-2α (MIP-2α) levels and greater neutrophil-driven inflammation following experimental infection by *H. pylori* [93,94,95,96]. Interestingly however, while *H. pylori* infection of male *Muc1^−/−^* mice was associated with increased stomach bacterial colonization compared with *Muc1^−/−^* female mice, *Muc1* null male mice had reduced gastric inflammation relative to matching female mice [97]. Subsequent mechanistic studies revealed that Muc1 diminished *H. pylori* colonization of the stomach both by steric hindrance and by acting as a releasable decoy receptor [98]. *MUC1* silencing by siRNA transfection of in vitro cultures of human gastric epithelial cells increased phosphorylation and nuclear translocation of NF-κB, and augmented IL-8 production compared with a nontargeting control siRNAs [94,96]. Conversely, ectopic over-expression of MUC1 in gastric epithelial cells decreased *H. pylori*-stimulated IL-8 production compared with MUC1-expressing cells. In *H. pylori*-infected gastric epithelia, MUC1 associated with β-catenin and the cytotoxin-associated gene A (cagA) protein, a major bacterial virulence factor [95]. Further, *H. pylori* infection of these cells increased nuclear translocation of β-catenin, and MUC1 over-expression decreased bacteria-driven β-catenin nuclear localization. MUC1 expression also inhibited gastric epithelial cell inflammatory signaling in response to a nucleotide-binding oligomerization domain (NOD) agonist as well as ligands for TLR3, 4, 5, 7, 8, and 9 [96]. Further studies demonstrated that Muc1 expression by mouse peritoneal macrophages negatively regulated the NLR family, pyrin domain-containing 3 (NLRP3) inflammasome through inhibition of TLR-dependent inflammasome priming, thereby suppressing gastric inflammation and reducing mortality in *H. pylori*-infected mice [99,100].

## 12. Role of MUC1 in Non-Pneumonia Respiratory Diseases

MUC1 may play an anti-inflammatory role in diseases other than bacterial pneumonia. Krebs von den Lungen-6 (KL-6) is a serum antigen comprised of a sialylated sugar chain of the MUC1 ectodomain [101]. Circulating KL-6 has been shown to be a sensitive biomarker of idiopathic pulmonary fibrosis [102], and a novel disease marker in adolescents and adults with cystic fibrosis (CF) [103]. Elevated serum KL-6 concentrations also were observed in children with acute exacerbations of asthma [104]. MUC1 levels in sputum were increased in adults with chronic obstructive pulmonary disease [105,106]. While murine Muc1 was not identified as a major component of the intestinal mucus in a mouse model of CF, Muc1 expression at both the mRNA and protein levels was increased in CF mice compared with disease-free controls [107,108].

MUC1 is aberrantly overexpressed by cancer cells, including lung carcinomas, where its overexpression is associated with poor survival [109,110,111]. In vivo and in vitro studies have established a variety of mechanisms through which MUC1 plays an oncogenic role in lung carcinogenesis [31]. MUC1 overexpression by airway epithelial cells contributed to cell transformation induced by exposure to cigarette smoke (CS) or CS-derived carcinogens through potentiation of EGFR-mediated signal transduction, destabilization of cell–cell adherens junctions, and loss of apical-basal polarity [112,113]. In CS-induced loss of apical-basal polarity, MUC1 redistributed over the entire plasma membrane and associated with EGFR, culminating in phosphorylation of the MUC1-CT [113]. Phosphorylated MUC1-CT interacted with the adherens junction proteins, β-catenin, and p120-catenin, leading to disruption of E-cadherin/β-catenin and E-cadherin/p120-catenin complexes, abrogation of cell–cell adhesion, and promotion of metastasis [114]. Further, exposure of polarized human bronchial epithelial cells to CS increased shedding of the highly glycosylated MUC1 ectodomain and concomitantly increased interaction of the membrane-bound MUC1-CT subunit with EGFR, c-Src, and p120-catenin [115]. The subsequent nuclear entry of p120-catenin in complex with MUC1-CT has been shown to be involved in epithelial-to-mesenchymal transition in the context of lung carcinogenesis [116]. In vivo and in vitro studies have also demonstrated that exposure of alveolar macrophages to CS increased MUC1 expression, leading to greater TNF release and TNF-driven increases in MUC1 expression by airway epithelial cells [117]. Taken together, these results suggest that CS-induced MUC1 overexpression, redistribution, phosphorylation, and protein–protein interactions promote lung carcinogenesis and tumor metastasis.

In non-small cell lung cancer (NSCLC) cells, MUC1 overexpression was associated with enhanced proliferation [118] and increased proangiogenic activity through activation of the PI3K-AKT pathway [109]. Treatment of NSCLC cells with agents that directly target the MUC1-CT suppressed the oncogenic function of MUC1. For example, GO-203—a 7-mer, cell-permeable, D-amino acid synthetic peptide derived from the MUC1-CT [119]—blocked MUC1-CT association with the p85 subunit of PI3K, induced reactive oxygen species-mediated death of NSCLC cells in vitro, and promoted regression of NSCLC tumor xenografts in nude mice in vivo [118]. In addition, the MUC1 inhibitory peptide, PMIP, that contains the β-catenin/p120-catenin binding motif present in the MUC1-CT, blocked CS-induced interaction between MUC1-CT and p120-catenin, thus stabilizing adherens junctions and decreasing cancer progression [116,120]. In spite of these finding, a recent study demonstrated that *Muc1* knockout mice exposed to nicotine-derived nitrosamine ketone (NNK), a CS-derived carcinogen, also known as 4-(methylnitrosamino)-1-(3-pyridyl)-1-butanone, had increased levels of the EGFR ligand, epiregulin, in lung tissues, augmented activation of the EGFR-AKT pathway, and heightened lung tumor multiplicity, compared with *Muc1*-expressing mice [121]. Further, *Muc1*-deficient mice, or treatment of A549 lung cancer cells with the MUC1-CT inhibitory synthetic peptide, GO-201 [122], increased epiregulin production by the cells [121]. Recognizing that their results were contrary to the well-accepted role of MUC1/Muc1 in oncogenesis, the authors suggested that Muc1 deficiency might increase epiregulin production to activate the EGFR pathway, thereby compensating for Muc1 loss in epithelial cells and promoting the EGFR-AKT pathway for lung tumorigenesis.

## 13. Perspectives

Although the role of MUC1 mucin in the airways remains to be completely elucidated, the recent reports that a human pathogen, *P. aeruginosa*, can stimulate MUC1-CT tyrosine phosphorylation suggests a potential role for this mucin in the host inflammatory response to microbial infection, specifically the resolution phase of inflammation through its ability to inhibit TLR signaling. Given that similar findings have been reported for other pathogens infecting respiratory tissues, it is likely that these effects are general in nature, although particular mechanistic details may differ. It is intriguing to speculate that genetic or epigenetic alterations of the MUC1-CT that block its ability to inhibit inflammatory signaling might contribute to the pathophysiology of chronic inflammatory lung diseases (e.g., chronic obstructive pulmonary disease, cystic fibrosis) and possibly hyperinflammatory disorders of other mucosal epithelia. Finally, it is worth noting that most of the mechanistic information about the anti-inflammatory role of MUC1/Muc1 during airway infection was based on in vitro studies mainly with epithelial cells. Therefore, future studies are warranted to understand the roles of MUC1 in non-epithelial cells, such as alveolar type II pneumocytes and fibroblasts during airway infection, and their potential contributions to inflammation and cancer.
